# Alterations in the blood‐based biomarker testing performances following correction for renal function

**DOI:** 10.1002/alz70856_097958

**Published:** 2025-12-24

**Authors:** Kenichiro Sato, Yoshiki Niimi, Ryoko Ihara, Atsushi Iwata, Kazushi Suzuki, Takeshi Iwatsubo

**Affiliations:** ^1^ The University of Tokyo, Bunkyo‐ku, Tokyo, Japan; ^2^ Unit for Early and Exploratory Development, The University of Tokyo, Bunkyo‐ku, Tokyo, Japan; ^3^ Tokyo Metropolitan Institute for Geriatrics and Gerontology, Itabashi‐ku, Tokyo, Japan; ^4^ National Defense Medical College, Tokorozawa, Saitama, Japan; ^5^ Department of Neuropathology, Graduate School of Medicine, The University of Tokyo, Bunkyo‐ku, Tokyo, Japan

## Abstract

**Background:**

Blood‐based biomarkers (BBMs), including plasma phosphorylated tau (*p*‐tau), have been expected as a promising, less‐invasive tool for detecting Alzheimer's disease (AD) pathology in real‐world applications. Plasma *p*‐tau levels are known to be elevated in individuals with chronic kidney disease (CKD), which may require caution when corrected for renal function since it alters testing performance – decreased sensitivity and increased specificity.

**Method:**

We first outline why correcting for renal function will alter testing performance, then examine its actual degree of alteration in sensitivity and specificity before and after the correction. Using plasma *p*‐tau217 data from our ongoing trial‐ready cohort study in Japan, we employed models with and without considering a renal function correction term to predict the amyloid PET scale, and differences in these models were assessed.

**Result:**

In the examined datasets, correction for renal function led to a decline in sensitivity by approximately 0.06, and an increase in specificity by approximately 0.09. Additionally, higher proportion of CKD in the examined samples was associated with a greater decrease in sensitivity and a greater increase in specificity.

**Conclusion:**

Our results demonstrated that while the deviations in performance metrics due to correction may be modest, they would not be negligible, particularly in cohorts with a higher proportion of CKD participants. This highlights the importance of establishing a robust correction equation for renal function to ensure the accurate real‐world application of BBMs. Furthermore, it may be recommended that when reporting BBM performance the proportion of CKD within the examined samples is also disclosed.

